# Supplementary figures and images for: Particulate matter collection by honey bees (Apis mellifera, L.) near to a cement factory in Italy
Source: PeerJ. 2018 Jul 24;6:e5322. doi: 10.7717/peerj.5322 (PMC6063219; doi:10.7717/peerj.5322)

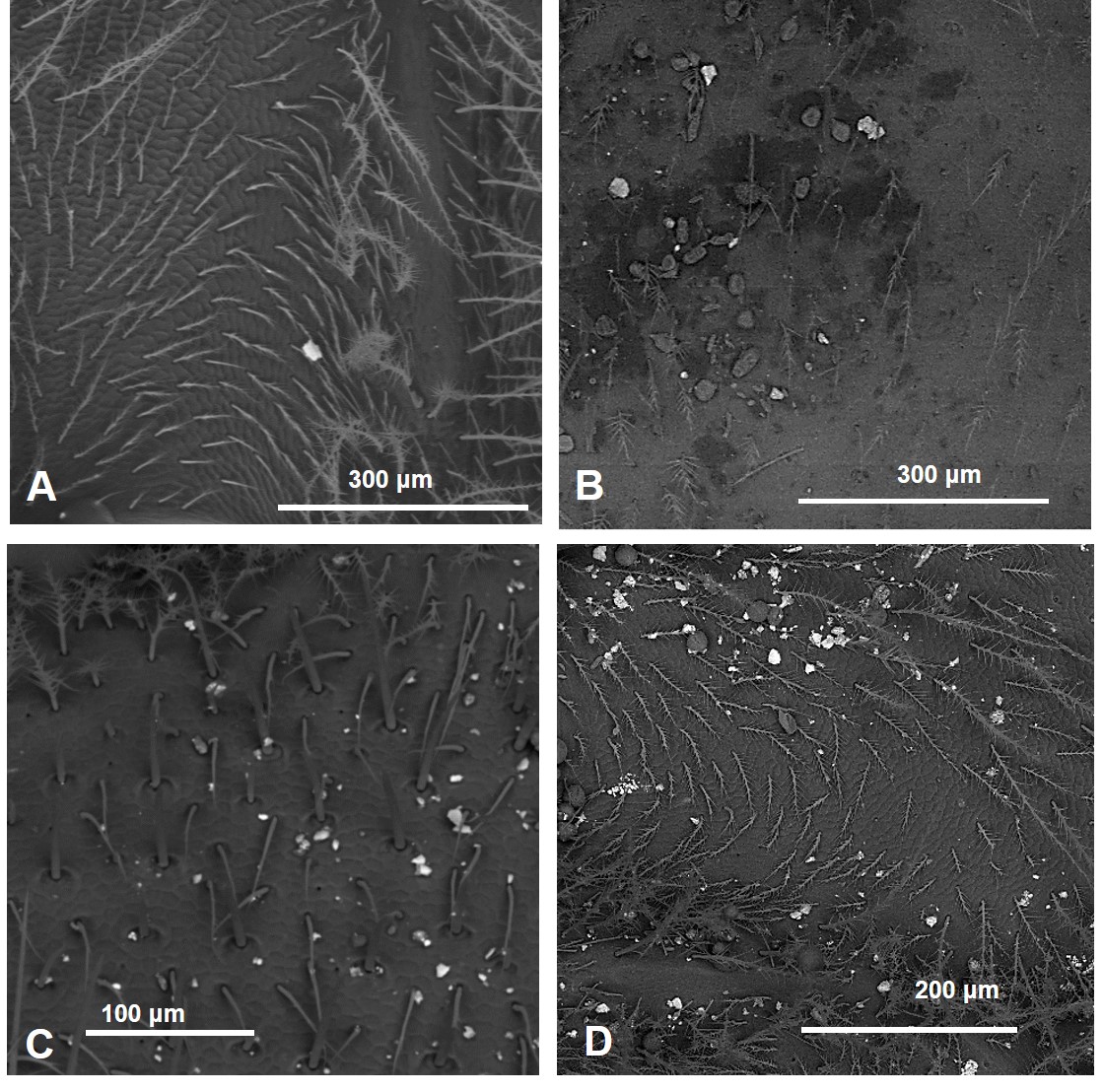

Supplement: Figure S1 — (A) scarcely/not contaminated;(B) fairly contaminated; (C) highly contaminated; (D) = heavily contaminated. [file peerj-06-5322-s001.jpg]

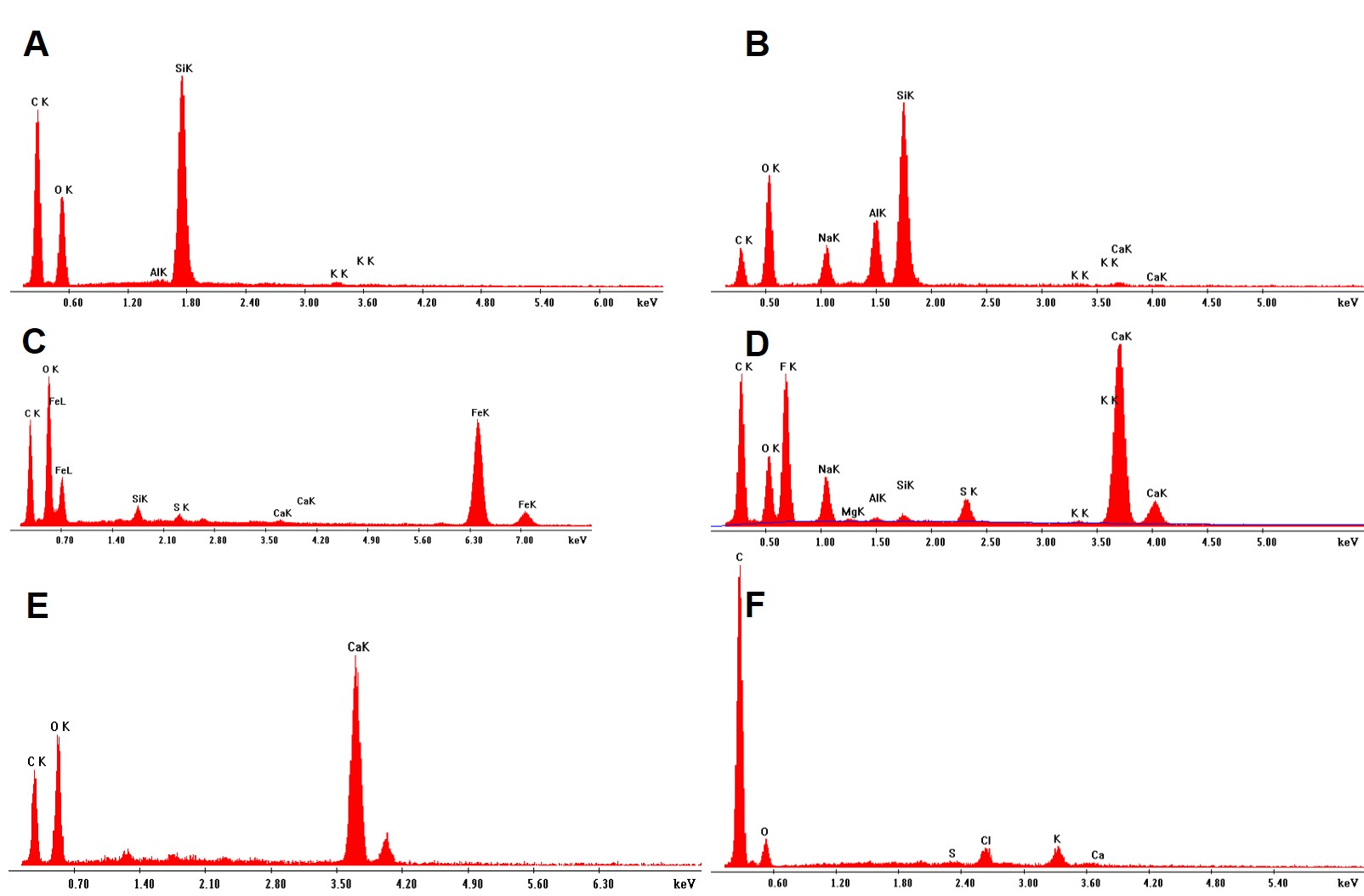

Supplement: Figure S3 — (A) Quartz, SiO4. (B) Plagioclase, NaAlSi3O8- CaAl2Si2O8. (C) Iron oxide/hydroxide. (D) Fluorite, CaF2. (E) Calcium oxalate, CaC2O4. (F) Sylvite, KCl. [file peerj-06-5322-s003.jpg]

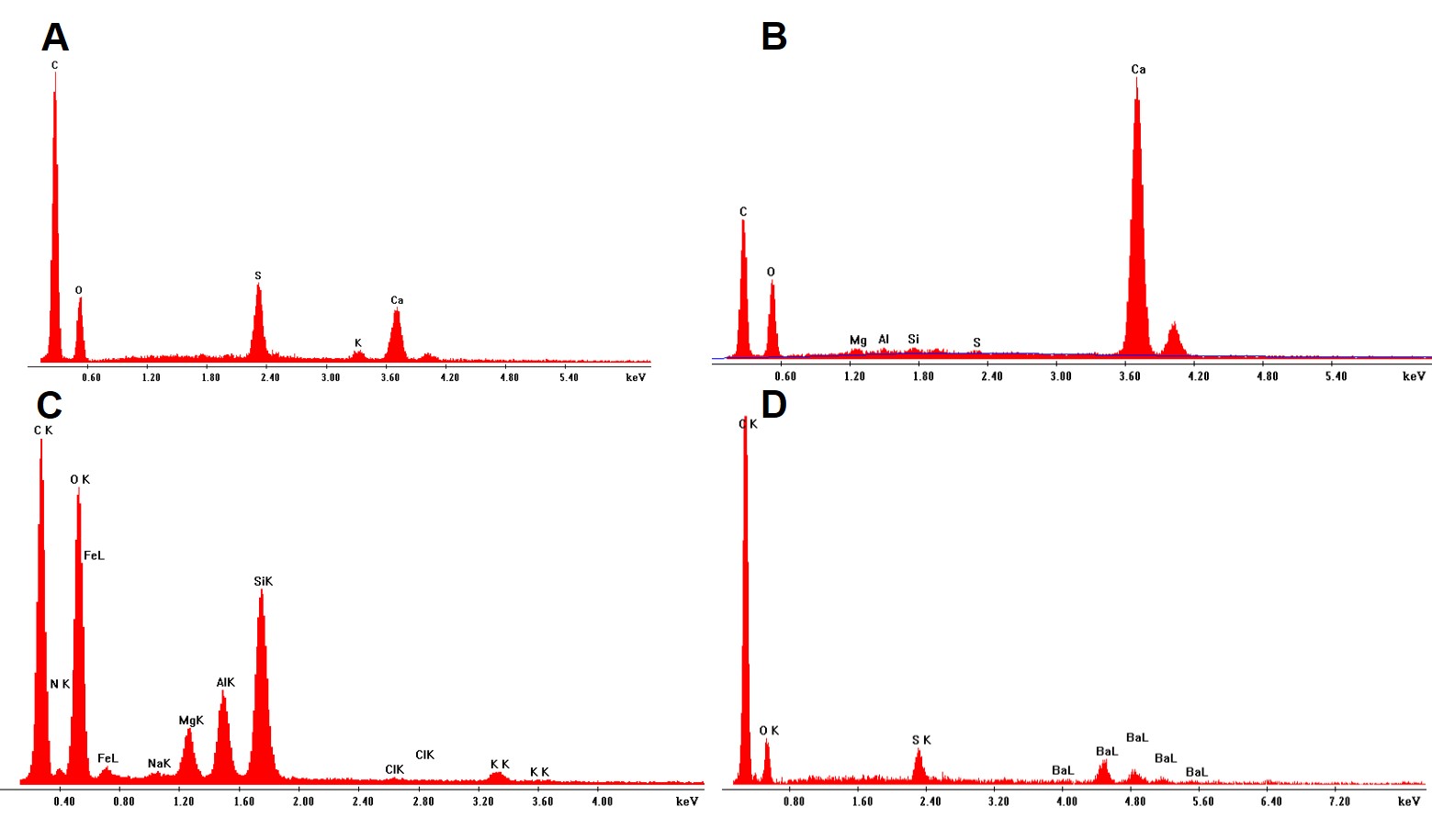

Supplement: Figure S4 — (A) Gypsum, CaSO4. (B) Calcite, CaCO3. (C) Chlorite mineral, (MgFeAl)8(SiAl)8O20(OH)16. (D) Baryte, BaSO4. [file peerj-06-5322-s004.jpg]
