# Supplementary material for: Particulate matter collection by honey bees (Apis mellifera, L.) near to a cement factory in Italy
Source: PeerJ. 2018 Jul 24;6:e5322. doi: 10.7717/peerj.5322 (PMC6063219; doi:10.7717/peerj.5322)
Supplement: Figure S2 — CP, bees living near the cement plant; CA, bees living far from the cement plant; W, fore-wings; H, heads [file peerj-06-5322-s002.docx]

|  | CP1 | CA1 | CP2 | CA2 | CP3 | CA3 | CP4 | CA4 | CP5 | CA5 |
| --- | --- | --- | --- | --- | --- | --- | --- | --- | --- | --- |
| W1 | 3 | 2 | 4 | 1 | 4 | 1 | 4 | 1 | 3 | 1 |
| W2 | 4 | 1 | 4 | 1 | 4 | 2 | 4 | 1 | 3 | 1 |
| W3 | 4 | 2 | 3 | 2 | 4 | 1 | 3 | 2 | 3 | 2 |
| W4 | 4 | 2 | 4 | 1 | 4 | 2 | 4 | 1 | 3 | 1 |
| H | 3 | 2 | 3 | 2 | 3 | 2 | 3 | 2 | 2 | 2 |

CP W1-4 *vs* CA W1-4, Mann-Whitney U test; p < 0.01, *significant*

CP H *vs* CA H, Mann-Whitney U test; p = 0.07, *not significant*
